# Combined frequency domain near-infrared spectroscopy and diffuse correlation spectroscopy system for comprehensive metabolic monitoring of inspiratory muscles during loading

**DOI:** 10.1117/1.JBO.29.3.035002

**Published:** 2024-03-26

**Authors:** Carlos A. Gómez, Laurent Brochard, Ewan C. Goligher, Dmitry Rozenberg, W. Darlene Reid, Darren Roblyer

**Affiliations:** a Boston University, Department of Biomedical Engineering, Boston, Massachusetts, United States; b St. Michael’s Hospital, Unity Health Toronto, Li Ka Shing Knowledge Institute, Keenan Research Centre, Toronto, Ontario, Canada; c St. Michael’s Hospital, Department of Critical Care, Toronto, Ontario, Canada; d University of Toronto, Interdepartmental Division of Critical Care Medicine, Toronto, Ontario, Canada; e University Health Network, Toronto General Hospital Research Institute, Toronto, Ontario, Canada; f University of Toronto, Department of Physiology, Toronto, Ontario, Canada; g University Health Network, Toronto General Hospital Research Institute, Ajmera Transplant Center, Toronto, Ontario, Canada; h University of Toronto, Division of Respirology, Temerty Faculty of Medicine, Toronto, Ontario, Canada; i University of Toronto, Department of Physical Therapy, Toronto, Ontario, Canada; j University of Toronto, Interdepartmental Division of Critical Care Medicine, Toronto, Ontario, Canada; k University Health Network, KITE – Toronto Rehabilitation Institute, Toronto, Ontario, Canada

**Keywords:** diffuse correlation spectroscopy, diffuse optics, mechanical ventilation, near-infrared spectroscopy, respiratory muscles

## Abstract

**Significance:**

Mechanical ventilation (MV) is a cornerstone technology in the intensive care unit as it assists with the delivery of oxygen in critically ill patients. The process of weaning patients from MV can be long and arduous and can lead to serious complications for many patients. Despite the known importance of inspiratory muscle function in the success of weaning, current clinical standards do not include direct monitoring of these muscles.

**Aim:**

The goal of this project was to develop and validate a combined frequency domain near-infrared spectroscopy (FD-NIRS) and diffuse correlation spectroscopy (DCS) system for the noninvasive characterization of inspiratory muscle response to a load.

**Approach:**

The system was fabricated by combining a custom digital FD-NIRS and DCS system. It was validated via liquid phantom titrations and a healthy volunteer study. The sternocleidomastoid (SCM), an accessory muscle of inspiration, was monitored during a short loading period in fourteen young, healthy volunteers. Volunteers performed two different respiratory exercises, a moderate load and a high load, which consisted of a one-minute baseline, a one-minute load, and a six-minute recovery period.

**Results:**

The system has low crosstalk between absorption, reduced scattering, and flow when tested in a set of liquid titrations. Faster dynamics were observed for changes in blood flow index (${\mathrm{BF}}_{i}$), and metabolic rate of oxygen (${\mathrm{MRO}}_{2}$) compared with hemoglobin + myoglobin (Hb+Mb) based parameters after the onset of loads in males. Additionally, larger percent changes in ${\mathrm{BF}}_{i}$, and ${\mathrm{MRO}}_{2}$ were observed compared with Hb+Mb parameters in both males and females. There were also sex differences in baseline values of oxygenated Hb+Mb, total Hb+Mb, and tissue saturation.

**Conclusions:**

The dynamic characteristics of Hb+Mb concentration and blood flow were distinct during loading of the SCM, suggesting that the combination of FD-NIRS and DCS may provide a more complete picture of inspiratory muscle dynamics.

## 1 Introduction

Mechanical ventilation (MV) is a lifesaving tool that has become ubiquitous in the intensive care unit (ICU) for critically ill patients with respiratory distress.^1^ Pre-COVID-19 pandemic rates of MV in the US were 2.7 episodes per 1000 population, and MV use was estimated to cost $27 billion per year.^2^ American hospitals reported a 31.5% increase in the number of MV cases during the COVID-19 pandemic.^3^ Despite the importance of MV in the ICU, it has several major physiological^4^^,^^5^ and psychological^6^^–^^9^ risks, including muscle disuse atrophy, ventilator induced diaphragm and lung injury, post-traumatic stress disorder, depression, anxiety, and cognitive impairments. Thus, it has been recommended that patients should be removed from MV at the earliest opportunity to minimize these risks, especially in older populations.^10^

The process of removing patients from MV, also known as the weaning, spans 40% of the duration of MV treatment^11^ and starts with a spontaneous breathing trial (SBT).^12^ During an SBT, a patient breathes with little to no assistance of a mechanical ventilator while physicians monitor a wide range of indices. These indices can be categorized as either subjective (i.e., subject displaying signs of pain or difficult breathing) or objective (i.e., heart rate and peripheral oxygen saturation).^13^ Current objective indices help to monitor the state of the patient during SBT, but these indices lack crucial insight into the metabolic state of the respiratory muscle themselves. This is unfortunate as the functional capacity of the respiratory muscles is key to the ability to sustain spontaneous breathing. Currently, there is no clinical standard to meet this need. Although electromyography (EMG) can monitor muscle activation (drive to breathe), it does not indicate the metabolic functional capacity required for spontaneous breathing. Moreover, clinically meaningful analysis of metabolic capacity is not available in real-time and thus is not evaluated as part of clinical practice during weaning. Therefore, there is a critical need for effective non-invasive technologies that can closely monitor patients’ respiratory muscles during weaning to guide the readiness and progression of the weaning process and to reduce the duration of MV.

Recently there have been initial investigations, including our own, into the use of near-infrared spectroscopy (NIRS) systems to monitor inspiratory muscles during various exercises in healthy subjects, with the stated goal of eventual use for patients on MV.^14^^,^^15^ NIRS is a non-invasive optical tool that can measure tissue hemoglobin and myoglobin concentrations via near-infrared light. These prior works have investigated the sternocleidomastoid muscle (SCM), a superficial accessory muscle that is recruited during elevated levels of ventilation, including respiratory distress.^16^ Although NIRS can give insight into the oxygen extraction of tissue, it does not provide a complete metabolic picture as it provides no information about the delivery of oxygen to tissue. When both oxygen extraction and blood flow are measured in unison, these two metrics can be combined via Fick’s principle to calculate the oxygen consumption rate, which may provide a more comprehensive metabolic profile of muscle function.^17^ In this work, we describe how we combined a custom frequency-domain NIRS (FD-NIRS) system with a custom diffuse correlation spectroscopy (DCS) system, which can measure blood flow, to evaluate metabolic changes of the SCM during a dynamic inspiratory loading protocol. Although there have been prior works combining NIRS and DCS systems to measure a range of tissues,^17^^–^^20^ this is the first to our knowledge to investigate dynamic changes in inspiratory muscles. It is also the first to characterize the unique alterations in oxygenation, blood flow, and oxygen extraction that occur during inspiratory muscle loading. These results provide an important foundation toward the use of combined NIRS-DCS in the ICU for patients on MV.

## 2 Methods

### 2.1 Custom Combined Diffuse Optical Spectroscopy and Diffuse Correlation Spectroscopy

**Fig. 1:**
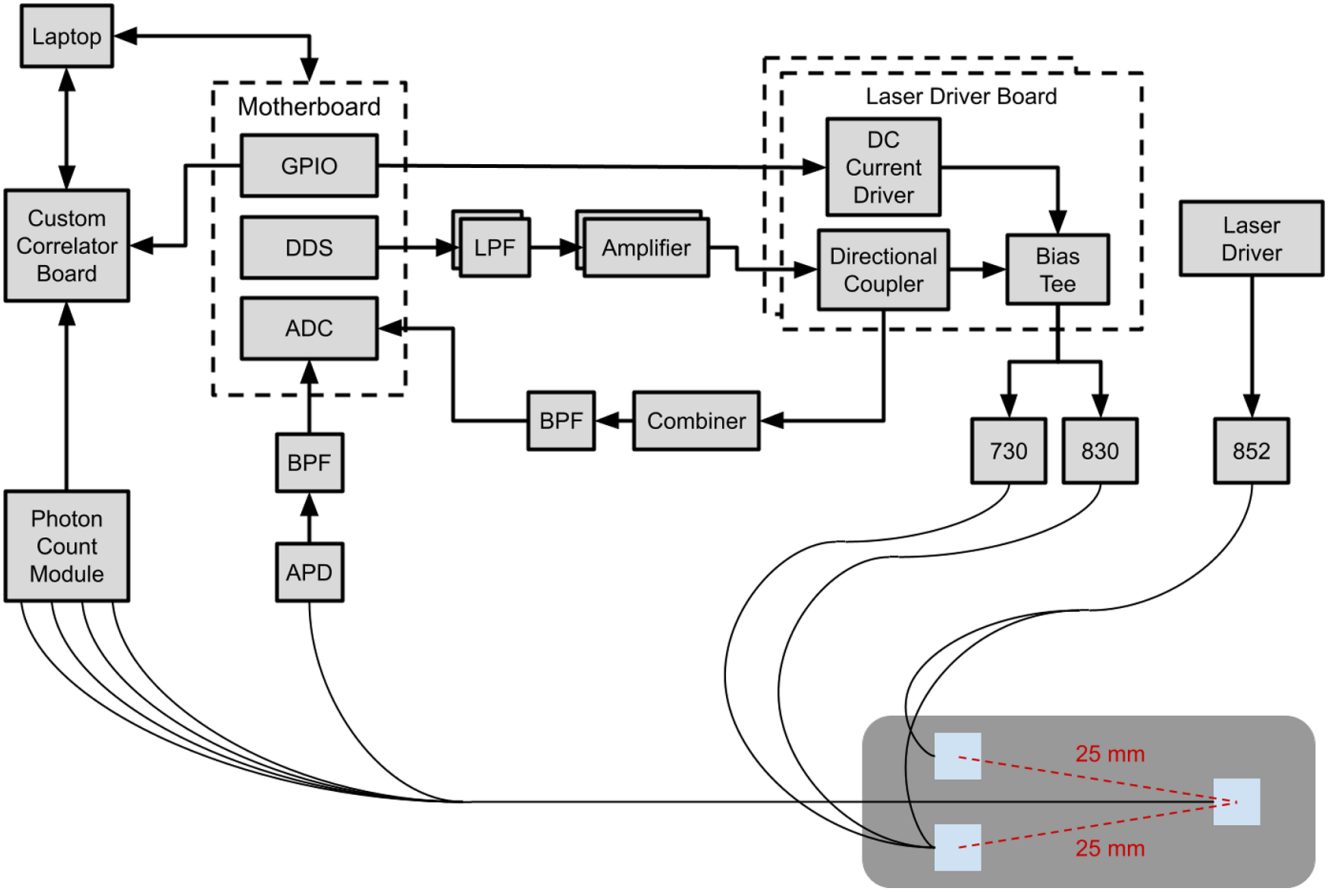
Block diagram of the custom combined FD-NIRS and DCS systems. Both systems are controlled by a single computer via separate custom software. The FD-NIRS system is composed of a custom motherboard that contains three daughter boards: an analog-to-digital converter board, a DDS board, and a general purpose input and output board. The DDS board outputs two sinusoidal signals that first travel through a lowpass filter with a frequency cutoff at 400 MHz, which then is amplified before being combined with the direct current (DC) via a bias tee. A reference signal for each FD-NIRS laser is transmitted to the ADC board by having a directional coupler send a signal to a combiner that combines both signals into one and goes through a bandpass filter with a frequency range of 110 to 180 MHz. The modulated signals with DC offsets drive the two lasers at two separate frequencies (139 and 149 MHz), which are optically coupled to the DCS’s long coherence laser by a custom probe. Once the FD-NIRS optical signal has traveled through the tissue, it is detected by an avalanche photon detector (APD) and goes through the same BPF as in the reference signal pathway before going to the ADC board. Additionally, the signal from the long coherence laser is detected by a photon module that contains four separate detectors and is sent to a custom correlator board that time stamps the signal. The systems are temporal multiplexed by having the FD-NIRS lasers modulated on and off, which is done via the GPIO board of the FD-NIRS. Additionally, the GPIO board sends a signal to the correlator board to time stamp when the FD-NIRS lasers are on. A custom probe with two source 3.5 mm prisms (blue squares) was used to split the DCS light source to increase the signal. Additionally, the probe only had a single detector 3.5 mm prism that co-localized both the NIRS and DCS signal.

A custom FD-NIRS system, which was previously used to monitor the SCM during repetitive quasi-isometric neck flexion in healthy volunteers,^15^ was integrated with a custom DCS system.^21^ Figure 1 shows a block diagram of the combined system and probe layout. The combined system has three fiber-coupled lasers co-localized via a custom dual source and single detector fiber probe. The FD-NIRS system has both a 730 and an 830 nm laser (Blue Sky FMXL730-030YFGA and Thorlabs LPS-830-FC), which are modulated by the direct digital synthesizers (DDS) at 139 and 149 MHz frequencies, respectively. The DCS system uses a long coherence laser (CrystaLaser DL852-100-S) with a wavelength of 852 nm. The DCS laser is coupled to a 105  μm core fiber (Thorlabs FG105LGA) with a numerical aperture (NA) of 0.22 that is split between two prisms, each 3.5  mm×3.5  mm, with one prism receiving 75% of the illumination power and the other receiving the remaining 25% of the illumination power. Each FD-NIRS laser is coupled to a separate 400  μm core fiber (Thorlabs FT400EMT) with NA 0.39. These fibers are coupled to the aforementioned prism, receiving 25% of the DCS illumination power. The use of dual prisms allows the illumination power to be distributed across a larger skin area, thus enabling overall higher illumination optical power while staying within American Standard Safety Institute limits. This method has been used previously.^22^^,^^23^ The two source prisms are separated by 8.5 mm and thus probe similar tissue regions as the SCM has an average width between 7 and 8 cm.^24^

An avalanche photodiode (APD) (Hammatsu S11519-30) is used as the detector for the FD-NIRS system and is coupled to a fiber bundle with NA 0.66. A single photon-counting module (Excelitas Technologies SPCM-AQ4C) is used for the DCS system and is fiber coupled to 125  μm core fiber (Thorlabs 780HP) with NA 0.13. Both fibers are coupled to the same 3.5  mm×3.5  mm detector prism. The source–detector separation of the custom probe is 25 mm. The systems were temporally multiplexed and had a sampling rate of 2 Hz, but down sampling (averaging of the G2 curves) was performed in post-processing to increase the signal-to-noise ratio of the DCS system, resulting in an effective sampling rate of 0.5 Hz for the combined system. Custom software was used to control both the FD-NIRS and DCS systems via the same laptop.

Tissue concentrations of oxygenated hemoglobin plus myoglobin (oxy [Hb+Mb]) and deoxygenated hemoglobin plus myoglobin (deoxy [Hb+Mb]) were determined using measurements from the FD-NIRS system. This was done by comparing the reference signal from the DDS and APD signal. Changes in the amplitude and phase induced by the tissue were calculated by a field programmable gate array in the digital FD-NIRS electronics. The information was then fed into a single layer look up table (LUT) to recover both absorption and reduced scattering coefficient ($\mu_{a}$ and $\mu_{s}^{\prime }$) of the tissue.^25^ The LUT was generated using Monte Carlo (MC) simulations that assumed an index of refraction of 1.37^26^ and an anisotropy value of 0.9.^27^ A calibration procedure was performed to remove the instrument response function.^28^ The recovered $\mu_{a}$ from both wavelengths was then fed into the Beer’s Law using known chromophore extinction coefficients to recover both Oxy [Hb+Mb] and Deoxy [Hb+Mb]. An assumption of 20% lipid fraction and 62.5% water fraction was used for the tissue.^29^^–^^32^ Total [Hb+Mb] was calculated by adding Oxy [Hb+Mb] and Deoxy [Hb+Mb] together. Tissue saturation (${S_{t}O}_{2}$) was then derived from oxy and deoxy [Hb+Mb] via the following equation:

$$S_{t}O_{2}=\frac{\mathrm{Oxy}[\mathrm{Hb}+\mathrm{Mb}]}{\text{Total}[\mathrm{Hb}+\mathrm{Mb}]}*100\%.$$

The blood flow index (${\mathrm{BF}}_{i}$) was determined using measurements from the DCS system. The custom correlator board time stamped the photon signal from the single photon counting module, and the signal was autocorrelated with itself over a small time period; this term is known as the intensity autocorrelation curve (g2). The g2 and the $\mu_{a}$ and $\mu_{s}^{\prime }$ from the FD-NIRS system were fed into a single layer LUT,^33^ generated using MC simulations that had an assumed index of refraction of 1.37^26^ and an anisotropy value of 0.9,^27^ to recover the ${\mathrm{BF}}_{i}$. The oxygen metabolic rate of (${\mathrm{MRO}}_{2}$) was then derived by the following equation based on Fick’s principle:

$${\mathrm{MRO}}_{2}={\mathrm{HGB}*\mathrm{BF}}_{i}*\frac{S_{p}O_{2}-S_{t}O_{2}}{\text{venous ratio}*\mathrm{mw}\;\mathrm{of}\;\mathrm{Hb}},$$

where HGB is the hemoglobin concentration of blood, ${\mathrm{SpO}}_{2}$ is the peripheral arterial oxygen saturation, the venous ratio is the proportion of blood volume in the venous circulation, and mw of Hb is the molecular weight of hemoglobin. The following assumptions were made for these parameters based on prior literature. HGB values of 14 and 16  g/dL were assumed for females and males, respectively,^34^ an ${\mathrm{SpO}}_{2}$ of 98% was assumed,^35^ a venous ratio of 0.75 was assumed,^36^ and the mw of Hb of 64,500  g/mol was assumed.^34^

### 2.2 Crosstalk Evaluation

Three different liquid phantom titrations were performed to assess the crosstalk between the three core measured parameters ($\mu_{a}$, $\mu_{s}^{\prime }$, and ${\mathrm{BF}}_{i}$). All titrations were performed in a container with the following dimensions: 150  mm×95  mm with 70 mm depth. The optical probe was placed directly on the surface of the liquid. For the absorption titration, an initial Intralipid solution of 0.5% lipid was created by diluting 20% stock Intralipid with deionized water. For each titration step, 0.48 mL of the batch solution was removed from and replaced with 0.48 mL of nigrosin solution with a 1.5  g/L concentration that was composed of nigrosin diluted in 0.5% Intralipid. Between each titration step, the solution was mixed for 60 s with an additional 90 s pause before measuring to ensure that there was only Brownian motion in the solution. Similarly, for the scattering titration, an Intralipid solution of 0.39% was created, and for each titration step, 2 mL was removed from the batch solution and 2 mL of 20% Intralipid was added. Again, the solution was mixed for 60 s with a 90 s pause before measuring. For the flow titration, a 0.35% Intralipid solution was used and constantly stirred with a magnetic stirrer at 64 rpm. Each titration step involved increasing the speed of the stirrer by 7 rpm and waiting for 60 s before measuring. Crosstalk was defined as the ratio of the normalized to baseline undesired change to the desired change expressed in decibels.

### 2.3 Healthy Volunteer Study

All measurements were conducted under an institutionally approved protocol (BU IRB 5618E). FD-NIRS and DCS measurements were conducted on 14 healthy volunteers (seven females and seven males) aged 26.3±1.4 years while they performed a breathing exercise with a respiratory device(s) (Philips Threshold IMT, POWERbreathe Plus IMT – Light Resistance, and POWERbreathe Plus IMT – Medium Resistance). First, subjects performed three maximum inspiratory pressure (MIP) tests, and their values were recorded from a pressure gauge (Vacumed 1505-120 Respiratory Pressure). The mean MIP was used to determine a high load (90% of MIP) and a moderate load (30% of MIP) for each subject. While the subjects were sitting upright, their right side SCM was located by having them look down and then to the left, which caused the SCM to be visible to an operator. The custom probe was placed over the SCM at approximately the center of the muscle while the subject was at a neutral head position; the specific location over the SCM was chosen to maximize the signal from the two instruments. Subjects performed two 8-min breathing exercises that consisted of 1 min for baseline, 1 min for load, and 6 min of recovery. The subjects breathed only through their mouth during baseline and recovery, and breathed through the respiratory device during the load phase. Subjects were given a 5 s count down before both the start and end of the load phase. Each subject performed a moderate load measurement first, and subjects were given a 10-min break before the start of the high load measurement.

### 2.4 Data Processing

**Fig. 2:**
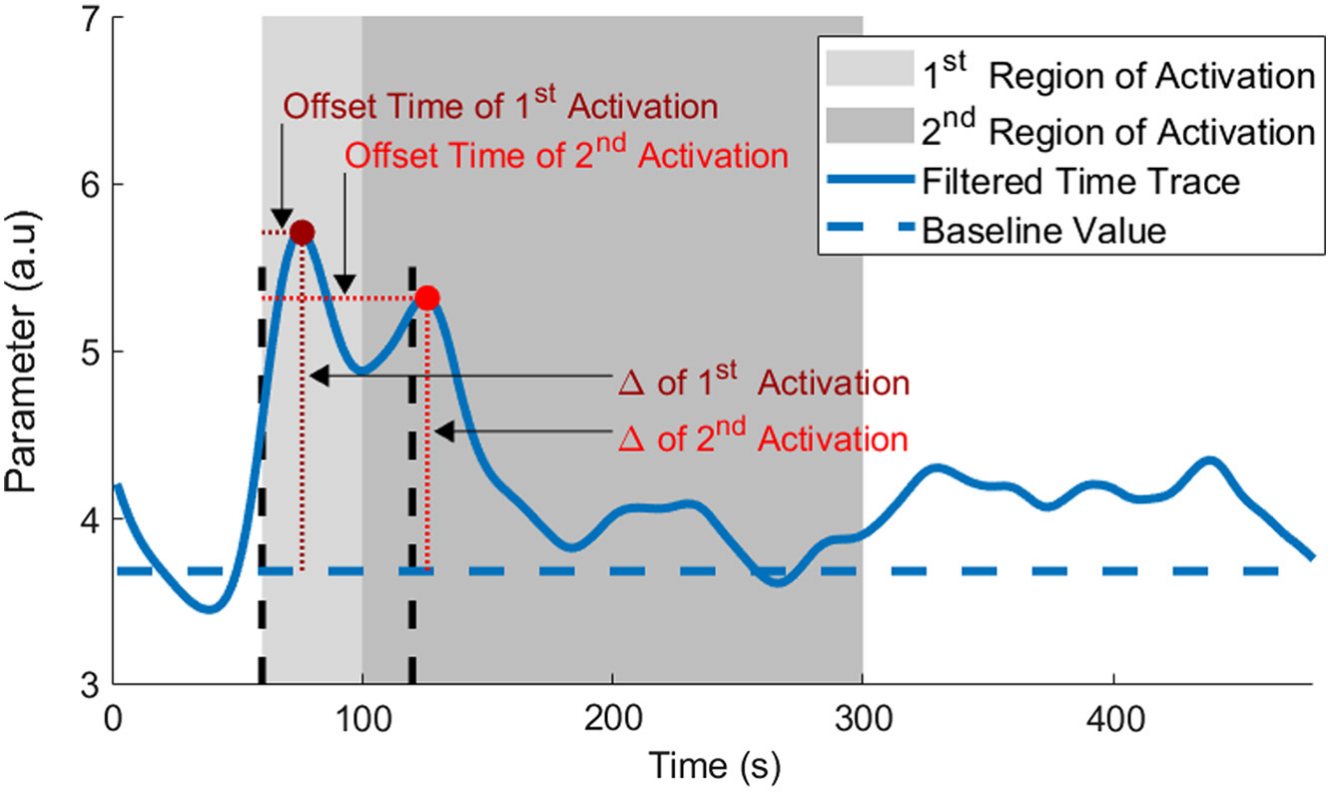
Example filtered time trace with offset and percent change from baseline (Δ) for the two regions of activation during the 8-min breathing exercise. Vertical black dashed lines indicate the start and end of the 1-min load portion of the respiratory exercise. The baseline value was determined by averaging the initial 50 s to avoid anticipatory effects. The offset time was determined by calculating the time difference from the event to the start of the load at the 60 s, which is indicated by the first vertical black dashed line. The percent change from baseline was calculated by measuring the difference from the event’s value to the baseline.

Time traces of six extracted parameters (${\mathrm{BF}}_{i}$, Oxy [Hb + Mb], Deoxy [Hb + Mb], Total [Hb + Mb], $S_{t}O_{2}$, and ${\mathrm{MRO}}_{2}$) were filtered through a second order Butterworth low pass filter with a frequency cutoff of 0.02 Hz to remove breathing oscillations. Offset time and percent change metrics were then extracted for both loads of each subject from the filtered time traces (Fig. 2). There was a wide range of responses to the load, with the most common being a double hump trace; thus two regions of activation were denoted. Offset was always calculated from the start of the load (t=60  s) due to the fact some subjects had only one peak that appeared in either region of activation. Figure 2 shows peaks during activation, but for some parameters (i.e., deoxy [Hb + Mb]), there was a decrease response, so the valleys were selected in those time traces. Baseline values were calculated by averaging the initial 50 s of the filtered time trace to avoid any anticipatory response. Normalization was performed by dividing the extracted parameter time traces by the baseline value. Respiration rates for baseline and both regions of activation were calculated by first filtering the time trace from the amplitude FD-NIRS signal at 730 nm with a second order Butterworth high pass filter with a frequency cutoff of 0.03 Hz. The filtered data was then used to find the mean time difference between peaks of each breath in a 30 s time window; this period between breaths was used to calculate breaths per minute by dividing 60 s by the period. Statistical analysis was done by running an unpaired two-tailed Student’s t-test to compare various extracted metrics (offset, normalized to baseline, the first 50 s, percent change, and respiration) between sex, load, and regions of activation. Systematical testing was done to determine if sex, load, and regions of activation had any statistically significant effects on the extracted metrics.

## 3 Results

### 3.1 Crosstalk

**Fig. 3:**
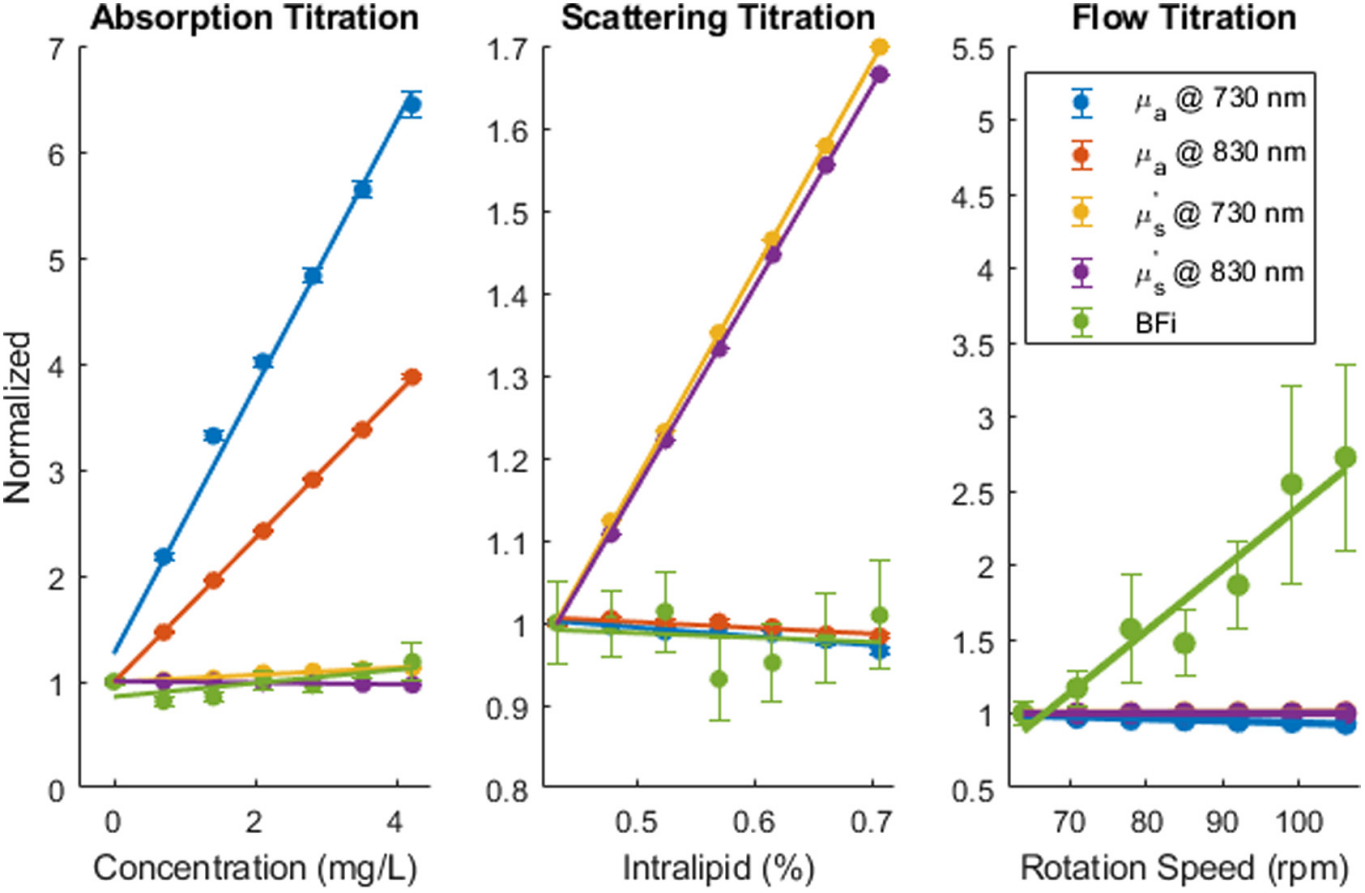
Normalized change from the baseline of $\mu_{a}$ and $\mu_{s}^{\prime }$ from the FD-NIRS system and ${\mathrm{BF}}_{i}$ from the DCS system during each of the three titrations. Error bars at each titration step represent the standard deviation. Solid line represents the best linear fit curve. Across all three titrations, there was low crosstalk between measure parameters. The absorption titration (left graph) had on average a 415% increase in $\mu_{a}$ and only on average a 5% increase in $\mu_{s}^{\prime }$ and a 19% increase in ${\mathrm{BF}}_{i}$. The scattering titration (center graph) had on average a 68.5% increase in $\mu_{s}^{\prime }$ and only on average a 2.5% in $\mu_{a}$ and a 1% increase in ${\mathrm{BF}}_{i}$. The flow titration (right graph) had a 276% increase in ${\mathrm{BF}}_{i}$ and on average a 3.5% decrease in $\mu_{a}$ and no change in $\mu_{s}^{\prime }$. All titrated parameters had a linear response during its titration as seen by the best fit trends. Both $\mu_{a}$ and $\mu_{s}^{\prime }$ had minor variations in values as seen by the small error bars, whereas ${\mathrm{BF}}_{i}$ had a larger variation.

The titration results are shown in Fig. 3. Each had a low (≤11  dB) crosstalk between the three measured parameters. For the absorption titration, absorption increased by 544% and 286% for 730 and 830 nm, respectively; reduced scattering increased by only 13% and −3% for 730 and 830 nm, respectively; and ${\mathrm{BF}}_{i}$ increased by 19%. For the scattering titration, reduced scattering increased by 70% and 67% for 730 and 830 nm, respectively; absorption decreased by only 3% and 2% for 730 and 830 nm, respectively; and ${\mathrm{BF}}_{i}$ increased by 1%. For the flow titration, ${\mathrm{BF}}_{i}$ increased by 276%; absorption changed by only −8% and 1% for 730 and 830 nm, respectively; and reduced scattering increased by 0% and 0% for 730 and 830 nm, respectively. Absorption and scattering had small variance in all three titrations, as seen by the error bars in Fig. 3. By contrast, ${\mathrm{BF}}_{i}$ had a larger variance, especially in the flow titration, which most likely arose from the increase in rpm of the stirrer. The increase in speed reduced the stability of the stirrer as larger fluctuations in rpm speed were noted at higher speeds, which likely explains the increase in error bar size at higher speeds shown in Fig. 3.

### 3.2 Healthy Volunteer Study

Baseline values were calculated for all six parameters for female and male participants, and the results are given in Table S1 in the Supplementary Material. Only oxy [Hb+Mb], total [Hb + Mb], and tissue saturation had significant differences in baseline values between the sexes with p values of 0.011, 0.018, and 0.044, respectively.

**Fig. 4:**
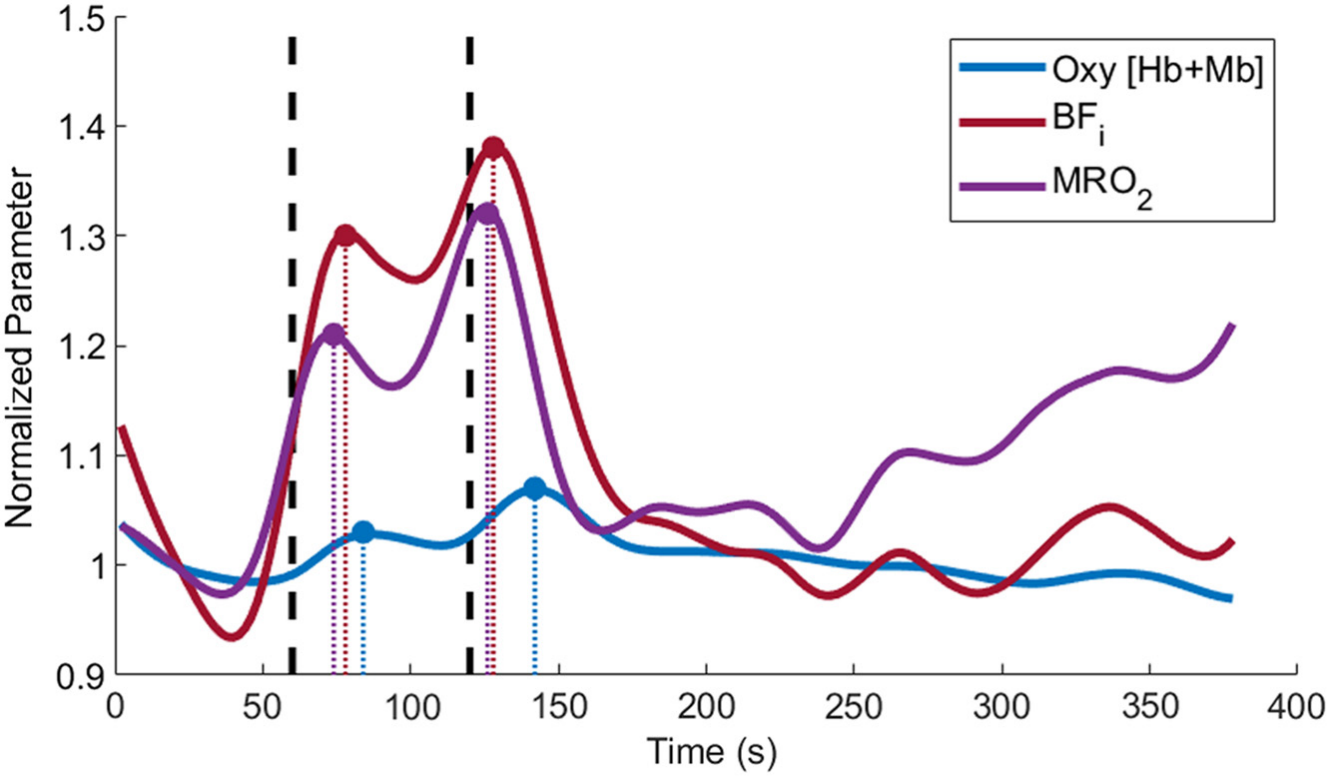
Time traces of the mean of all male data (n=7). Traces were normalized to baseline (i.e., the first 50 s). Vertical black dashed lines indicate the start and end of the 1-min load portion of the respiratory exercise. Data has the last 100 s removed due to one subject coughing, which resulted in large muscle activation. The time offset values (i.e., time from the start of the exercise, indicated by the first vertical dashed line), were typically shorter for ${\mathrm{BF}}_{i}$ and ${\mathrm{MRO}}_{2}$ compared with hemoglobin + myoglobin concentration and saturation changes. Additionally, the percent changes were typically larger for ${\mathrm{BF}}_{i}$ and ${\mathrm{MRO}}_{2}$ compared with hemoglobin + myoglobin changes.

Figure 4 shows the mean of all males (n=7) time traces for Oxy [Hb+Mb], ${\mathrm{BF}}_{i}$, and ${\mathrm{MRO}}_{2}$, which helps to highlight the most common features observed in the data. For example, the time offset values were typically shorter for ${\mathrm{BF}}_{i}$ and ${\mathrm{MRO}}_{2}$ compared with hemoglobin concentration and saturation changes. Additionally, the percent changes were typically larger for ${\mathrm{BF}}_{i}$ and ${\mathrm{MRO}}_{2}$ compared with hemoglobin changes. A double hump feature was commonly observed after the start of the load as shown by the two peaks in the measured parameters.

**Fig. 5:**
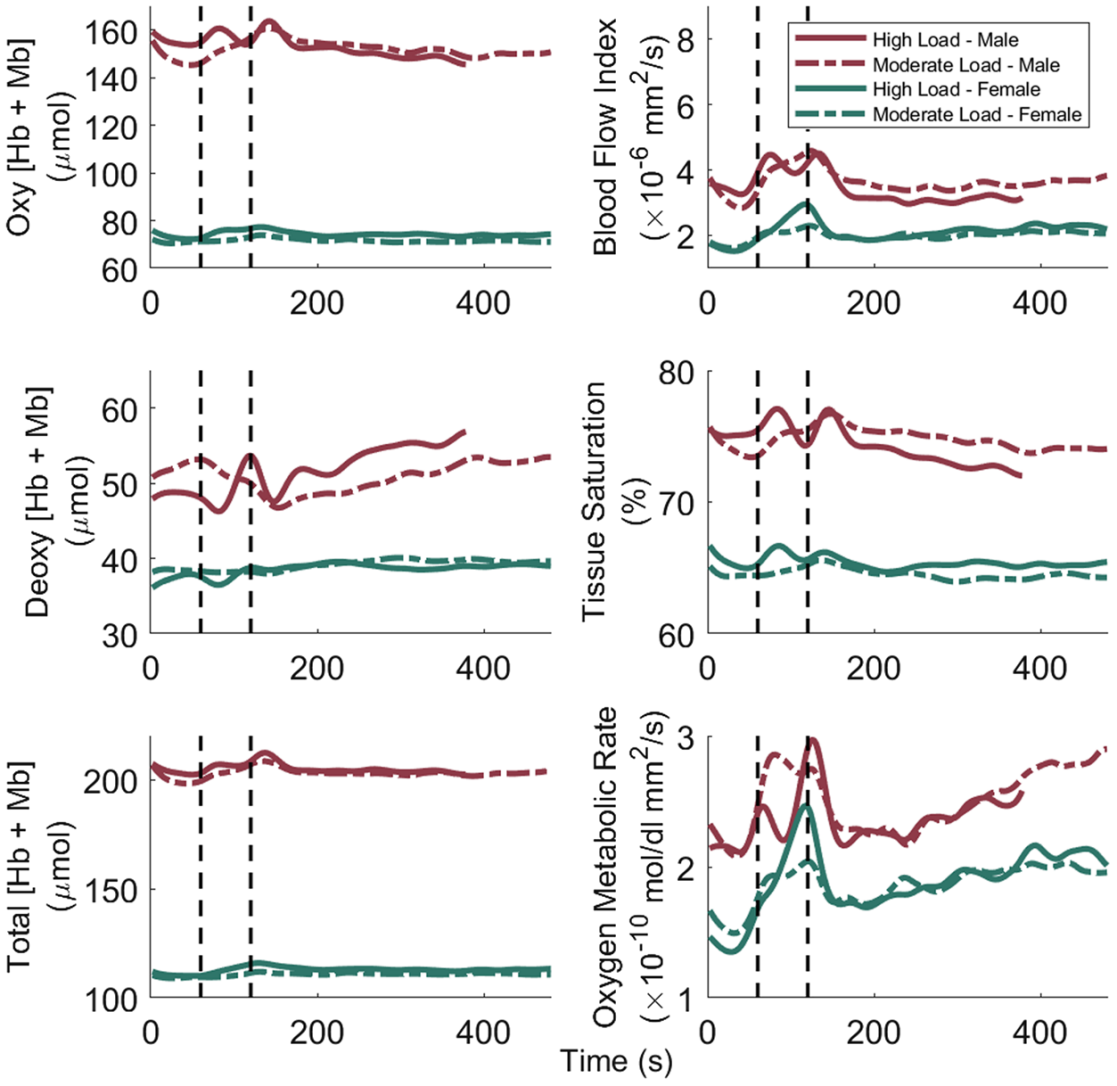
Mean filtered time traces of the six muscle parameters from the combined system. Vertical black dashed lines indicate the start and end of the one-minute load portion of the respiratory exercise. The high load, male time traces have the last 100 s removed due to one subject coughing, which resulted in large muscle activation. Only the oxy and total [Hb+Mb] had statistical differences between sexes due to large subject variances. The majority of mean time traces had a double perturbation event, with most having a positive change from baseline with only deoxy [Hb+Mb] having a negative change from baseline. The offset time of the second perturbation for ${\mathrm{MRO}}_{2}$ was significantly different between the sexes (p=0.03).

The mean filtered time traces for all subjects for the six parameters are plotted in Fig. 5, and all individual filtered time traces are show in Figs. 1–6 in the Supplementary Material. The time traces were separated by sex and by load, resulting in four mean time traces per subplot. Activation of the SCM was observed in all six metrics as indicated by changes from baseline after the start of the load, with deoxy [Hb+Mb] being the only metric to show a decrease from baseline, whereas the other five metrics had increases from baseline. Additionally, the mean time traces showed a clear double hump feature within the 240-s time window after the start of the load. These double features led us to extract offset and percent change for the two regions of activation for all six parameters. The mean and standard deviation for the offset for all sex and load combinations during both regions of activation are shown in Table S1 in the Supplementary Material. Additionally, the mean and standard deviation for the absolute change for all sex and load combinations during both regions of activation are given in Table 2 in the Supplementary Material. The rate of respiration for all sex and load combinations during baseline and both regions activation is given in Table S3 in the Supplementary Material. There was no significant difference in the respiration rate between sex, load, and region versus baseline.

**Fig. 6:**
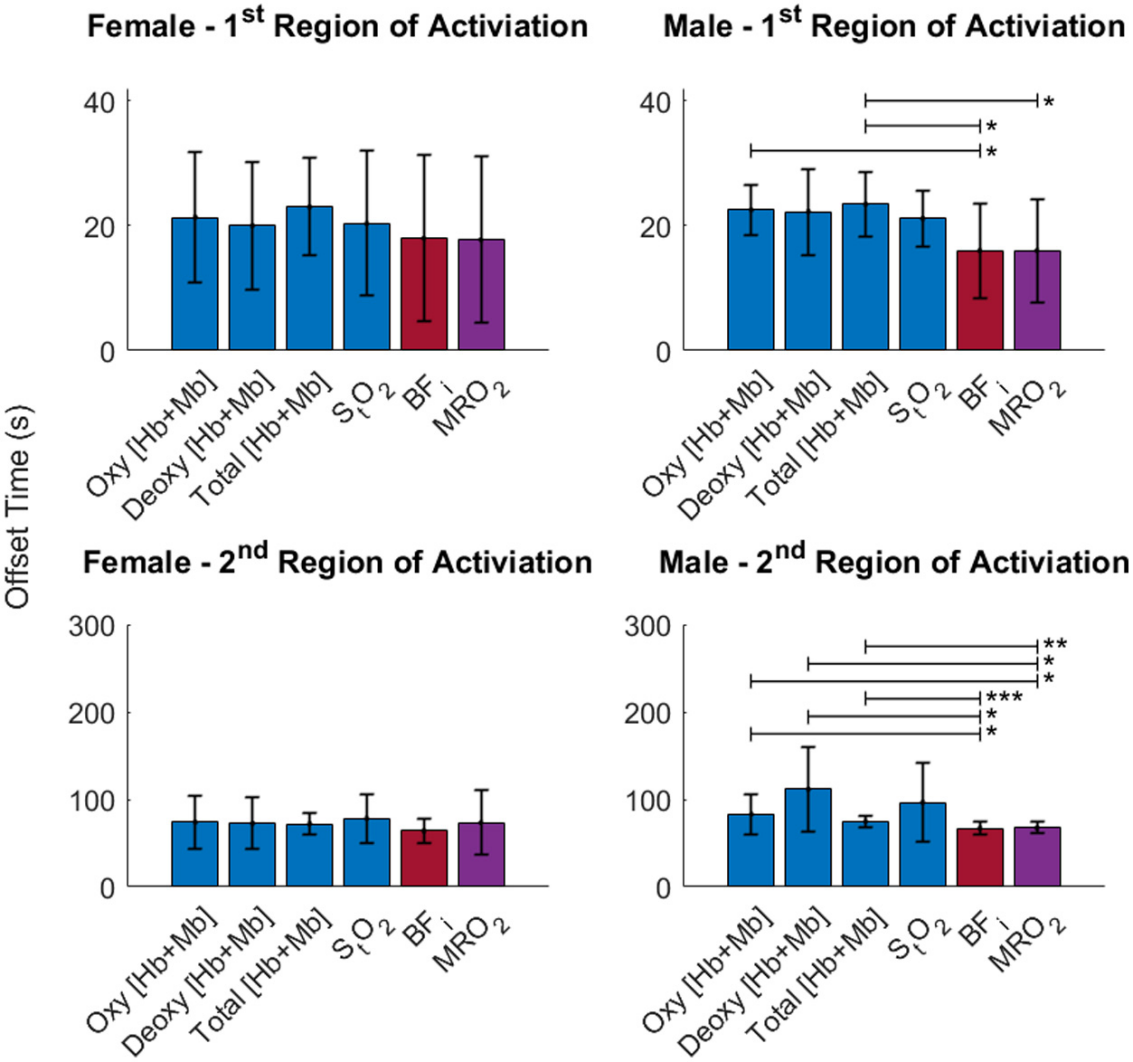
Offset values for the six muscle parameters. Both moderate and high loads are included. Males had significantly shorter offsets for both ${\mathrm{BF}}_{i}$ and ${\mathrm{MRO}}_{2}$ when compared with total [Hb+Mb], oxy [Hb+Mb], and deoxy [Hb+Mb]. There were no statistically significant differences for females. For males, these results suggest that ${\mathrm{MRO}}_{2}$ is tightly coupled with the ${\mathrm{BF}}_{i}$ temporal responses. *p<0.05, **p<0.01, ***p<0.001.

The offset values from the six muscle parameters were analyzed for each sex and region of activation to determine if there were any differences between the temporal dynamics of the six parameters (Fig. 6). The offset values were separated by sex and not by load due to the fact that there was no significant difference between the loads. There was, however, a significant difference between the male and female offsets (p=0.03) for the second region of activation of ${\mathrm{BF}}_{i}$ during high loads. Females showed no significant differences between the offsets of the six parameters for either region of activation. Males had significantly shorter offsets for both ${\mathrm{BF}}_{i}$ (p=0.043, p=0.045, and p=0.0008) and ${\mathrm{MRO}}_{2}$ (p=0.046, p=0.046, and p=0.009) in the second region of activation when compared with oxy [Hb+Mb], deoxy [Hb+Mb], and total [Hb+Mb].

**Fig. 7:**
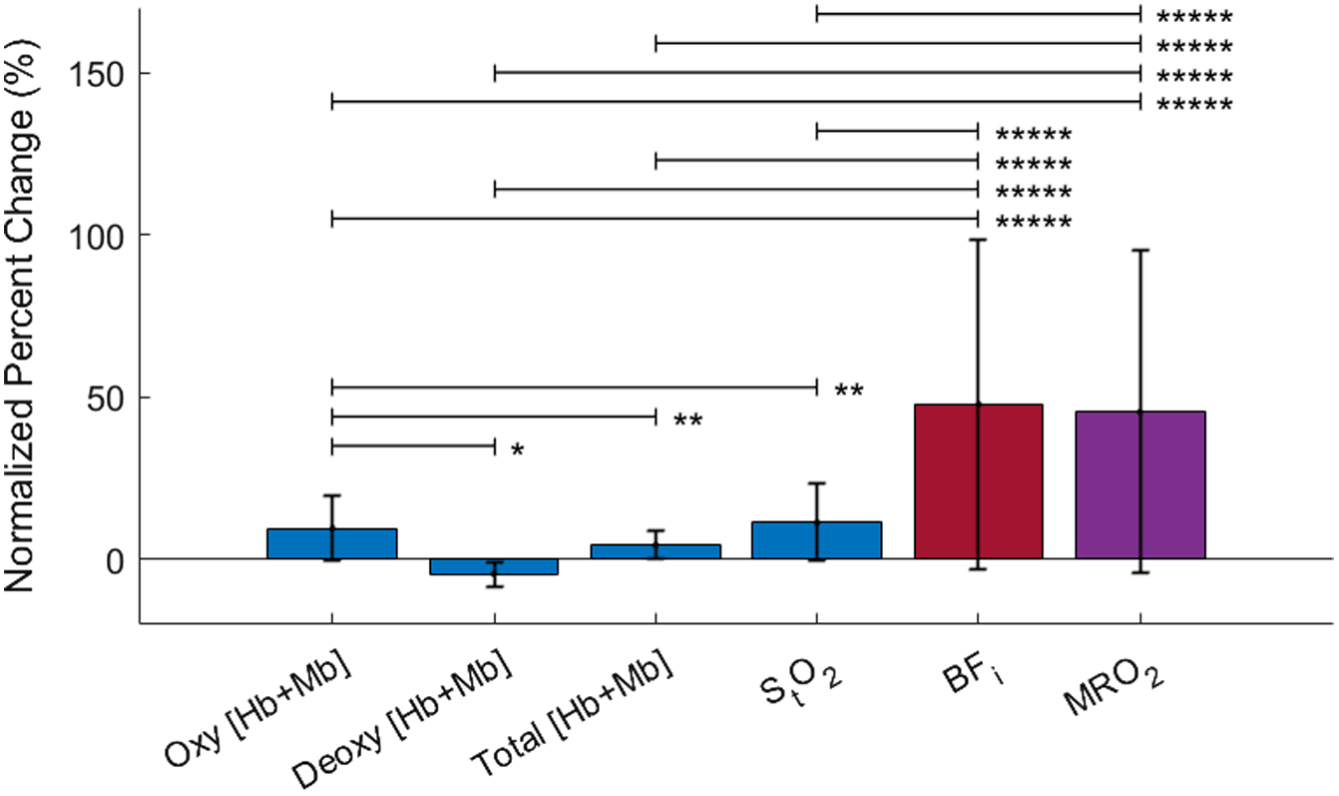
Percent change for the six muscle parameters when pooled across sex, region of activation, and load. Data was normalized to baseline (i.e., the first 50 s). ${\mathrm{BF}}_{i}$ and ${\mathrm{MRO}}_{2}$ had the largest percent increases. Deoxy [Hb+Mb] decreased, oxy [Hb+Mb] increased, and total [Hb+Mb] only increased slightly. An increase in tissue saturation was also observed. *p<0.05, **p<0.01, ***p<0.001, ****p<0.0001, *****p<0.00001.

Similar statistical analysis was performed on percent change of the six parameters (Fig. 7). Data was pooled as there was no significant difference between sex, load, or region of activation. ${\mathrm{BF}}_{i}$ and ${\mathrm{MRO}}_{2}$ had the largest increase compared with the other metrics. Additionally, oxy [Hb + Mb], total [Hb+Mb], and $S_{t}O_{2}$ had increases from baseline, whereas a decrease from baseline occurred in deoxy [Hb+Mb].

## 4 Discussion

We successfully combined a custom FD-NIRS system and custom DCS system to operate in unison for the purpose of dynamically monitoring the SCM during loading. The combined system was validated via three liquid titrations and a healthy volunteer study. The titrations showed minimal crosstalk in the measured parameters ($\mu_{a}$, $\mu_{s}^{\prime }$, and ${\mathrm{BF}}_{i}$) when swept across anticipated physiological ranges. Additionally, the changes in measured parameters (oxy [Hb+Mb], deoxy [Hb+Mb], total [Hb+Mb], ${\mathrm{StO}}_{2}$, ${\mathrm{BF}}_{i}$, and ${\mathrm{MRO}}_{2}$) from the SCM were continuously monitored during respiratory loading via a respiratory device. These parameters were analyzed to determine the typical physiological response of the SCM during a short perturbation load in young healthy subjects. Several key trends were observed, including shorter time offsets for ${\mathrm{BF}}_{i}$ and ${\mathrm{MRO}}_{2}$ compared with hemoglobin + myoglobin based parameters in males, and larger percent changes in ${\mathrm{BF}}_{i}$, and ${\mathrm{MRO}}_{2}$ compared with hemoglobin + myoglobin based parameters in both males and females. These data suggest that, at least in some circumstances, the dynamic characteristics of blood flow and oxygen extraction are substantially different during SCM loading compared with NIRS-based hemoglobin + myoglobin measures. This suggests that the use of combined FD-NIRS and DCS may provide a more complete picture of inspiratory muscle dynamics, potentially assisting in the evaluation of patients under MV in the future.

The key finding that ${\mathrm{BF}}_{i}$ and ${\mathrm{MRO}}_{2}$ had larger changes than hemoglobin + myoglobin based parameters was also observed in a prior study during the initial response of a quadriceps exercise.^17^ This, combined with the fact that ${\mathrm{BF}}_{i}$ and ${\mathrm{MRO}}_{2}$ had faster offset times compared with hemoglobin + myoglobin based parameters in males, is consistent with the hypothesis that a change in oxygen delivery (through increased blood flow via vasodilation mechanisms), rather than a change in oxygen extraction, is the more rapid and dominant metabolic response mechanism for the type of inspiratory perturbation evaluated here. It is of note that larger changes in oxy [Hb+Mb] and deoxy [Hb+Mb] have been reported when using a standalone NIRS system when measuring the SCM.^14^^,^^15^ This difference may be due to the different type of muscle activation that recruited more motor units due to higher loads^14^^,^^15^ and longer duration^14^ in this study compared with the prior work. This study used a short one-minute load, whereas the prior studies loaded the SCM until failure. Short activation, like the one-minute load used here, might be more feasible as a means to evaluate readiness for weaning in an ICU setting as it is more rapid and may be less likely to cause respiratory muscle fatigue or damage. Additionally, the short load period in this study contributed to there being no statistical significance between the moderate and high loads. There was no evidence to show that the subjects had reach their max oxygen consumption rate as the ${\mathrm{MRO}}_{2}$ did not show signs of plateauing during either region of activation, which could have led similar temporal and percent change values between loads.

This study showed some differences between the sexes. This is consistent with previous work that has shown differences in respiratory system mechanics during activation between the sexes.^37^ Baseline values in oxy [Hb+Mb] and total [Hb+Mb] were different between the sexes, with males having higher concentrations on average, which has also been reported by other groups.^15^^,^^19^^,^^38^ This may be attributable to the known hemoglobin content difference between sexes.^39^ There were also some sex dependent responses in temporal offset of some parameters. For example, during high loads in the second region of activation, statistical differences were observed in offset in ${\mathrm{BF}}_{i}$ between the sexes. Overall males had more rapid ${\mathrm{BF}}_{i}$ and ${\mathrm{MRO}}_{2}$ activation in both regions of activation, whereas females did not have any single parameter change faster than the others. These offset trends between the sexes are somewhat difficult to interpret as the results could be impacted by several factors: short load period, anticipatory response, and subject variance in respiratory muscle group response.

The mean time traces showed a unique double hump in all six-muscle metrics, although there was significant variance between subjects. This variability most likely arises from the fact that SCM is only a single muscle in a group of muscles that work together to allow inspiration to occur. When faced with a load, the body might respond by activating other muscles of inspiration including the diaphragm, intercostals, and scalene muscles, in various proportions alongside the SCM. Further work should investigate the subject variance response by probing various respiratory muscles during similar loads. Additional work should investigate the respiratory response of the SCM during extended respiratory loading.

We showed here that the SCM oxygen consumption is driven by an increase in blood flow for a short period of load, but this might not be the case for extended loads. Work with FD-NIRS and DCS on the quadriceps during exercise has shown that blood flow dominates the early stages of muscle activity, but drops in tissue saturation do occur near the muscle failure point.^17^ The SCM most likely responds in a similar manner, and this could be insightful for comparing between healthy subjects and patients on MV with or without established respiratory muscle weakness.

This study has several important limitations. First, DCS is known to be sensitive to motion artifacts including those that occur during muscle contraction.^17^ Muscle movement was observed in a portion of the study subjects, which may have affected the results. This concern is mitigated by the fact that there were large increases in ${\mathrm{BF}}_{i}$ across all subjects, even those that did not have visible muscle contractions during loading. In the future, an accelerometer could be incorporated into the probe to help characterize movement. An additional potential issue is related to the dual source illumination configuration. Although this strategy helps increase overall illumination power and the quantity of detected photons by the DCS system while meeting ANSI safety standards, it is possible that the 75%/25% configuration has the potential to alter the g2 measurements in an unanticipated manner or introduce unwanted noise in the measurements due to the uneven split. It is of note that other groups have used a 50%/50% configuration.^22^^,^^23^ Another limitation of this study is the use of a simple homogeneous inverse model. In the future, a more complex multi-layer model that accounts for skin, adipose, and muscle layers may provide more accurate extractions of SCM properties. Finally, young healthy volunteers were measured in this study, and measurements may not be representative for older subjects or subjects with medical conditions. Future studies should include a more varied subject pool that accounts for age and medical conditions (asthma, obesity, diabetes, etc.). Despite these limitations, the study suggests that the combined methodology of FD-NIRS and DCS may be useful in characterizing inspiratory muscle response during weaning from MV, with the eventual goal of noninvasive identification of patients who are ready for weaning. Additionally, the technique might also play a key role in timing of intubation for MV treatment. Further study in healthy volunteers and patients on MV is warranted.

## 5 Conclusion

Custom FD-NIRS and DCS devices were combined to continuously monitor the SCM during a one-minute respiratory exercise task. There were minor differences between the sexes in some baseline parameters. Importantly, the proportional increases in BFi and ${\mathrm{MRO}}_{2}$ were greater than changes in hemoglobin + myoglobin based parameters for all subjects, and the temporal dynamics of BFi and ${\mathrm{MRO}}_{2}$ were faster in males compared with hemoglobin + myoglobin based parameters. These trends suggest that metrics measured with FD-NIRS and DCS have distinct dynamics during loading in the SCM, and therefore it may be beneficial to utilize both technologies in the future when monitoring patients on MV.

## Supplementary Material



## Data Availability

Scripts, data, and associated instructions for performing each step of the analysis from raw data up to filtered time traces of each subject are provided in a repository on Github: https://github.com/BU-BOTLab/FDNIRS_paper_JBO2023. Furthermore, the exact time points and perturbation values from all parameter for each subject are included in the repository. Statistical analysis is performed on their recorded values, and further information can be provided upon request.
