# Supplementary material for: Combined frequency domain near-infrared spectroscopy and diffuse correlation spectroscopy system for comprehensive metabolic monitoring of inspiratory muscles during loading
Source: J Biomed Opt. 2024 Mar 26;29(3):035002. doi: 10.1117/1.JBO.29.3.035002 (PMC10965138; doi:10.1117/1.JBO.29.3.035002)
Supplement: Supplementary file 1 [file JBO_029_035002_SD001.pdf]

Supplement

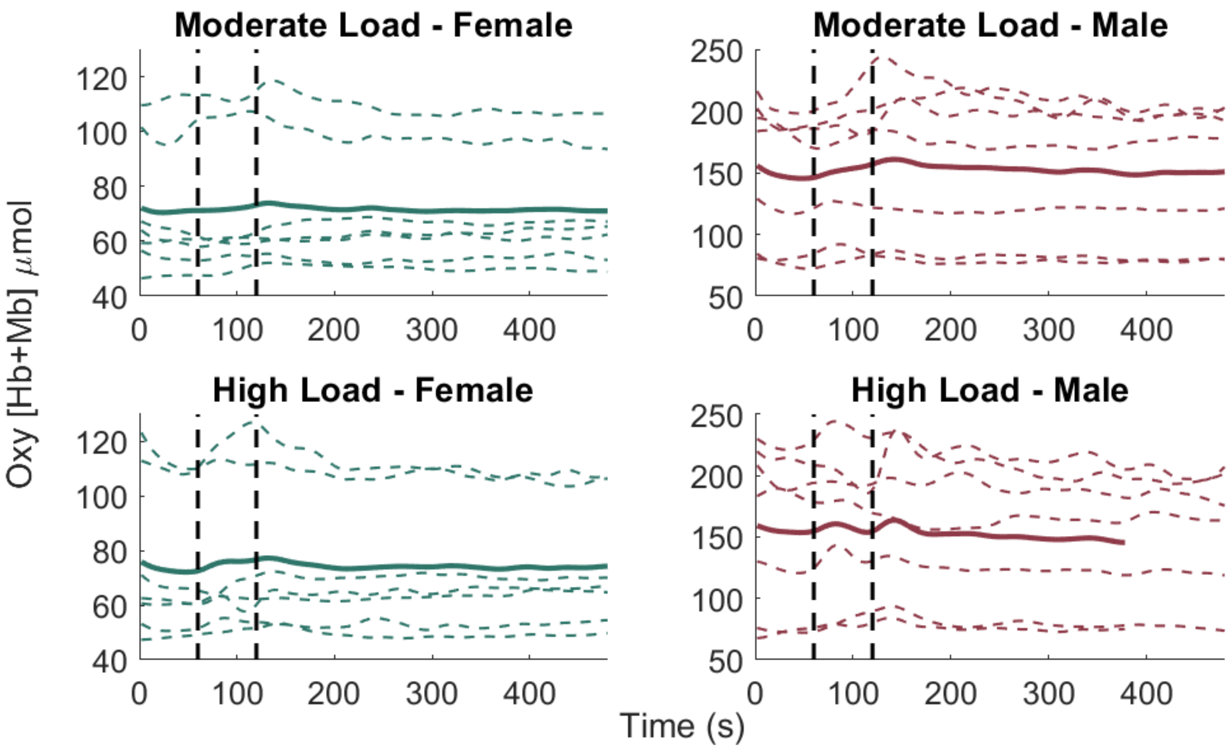

Figure S1. Filtered time traces of each subject and mean filtered time trace for each load and sex. Vertical black dashed lines indicate the start and end of the one-minute load portion of the respiratory exercise. The high load, male time trace has the last 100 seconds removed due to one subject coughing which resulted in large muscle activation.

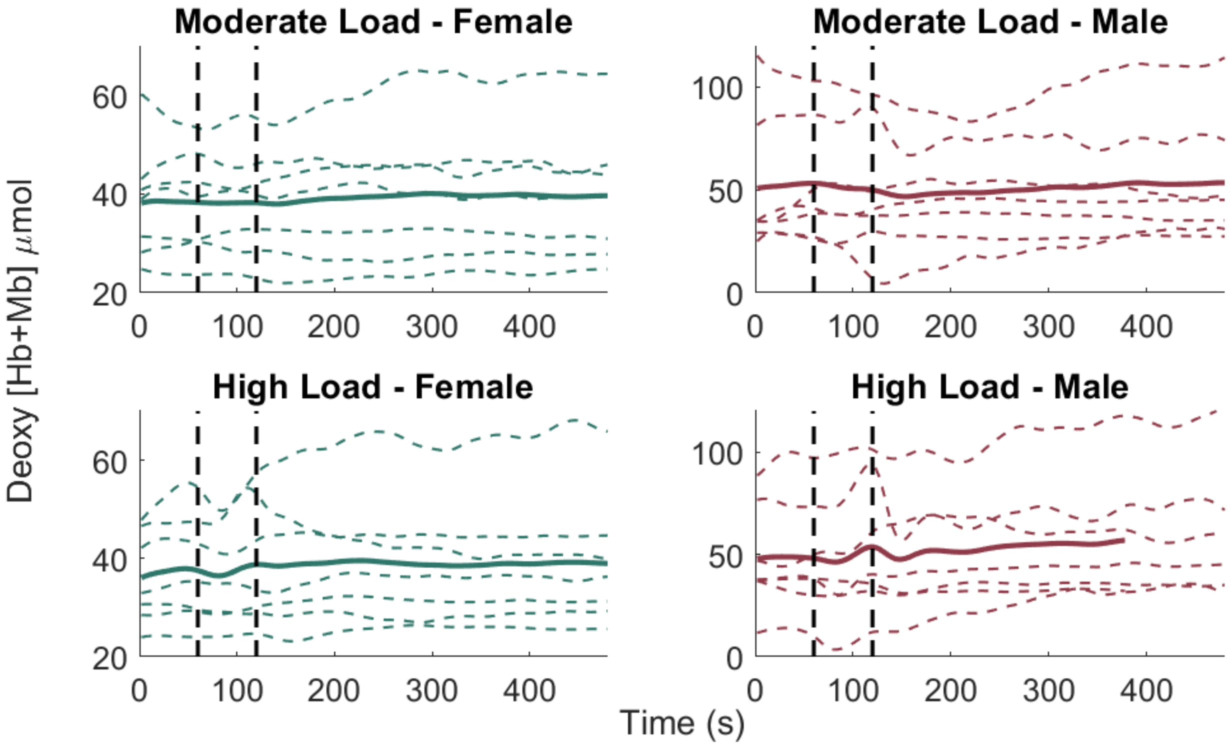

Figure S2. Filtered time traces of each subject and mean filtered time trace for each load and sex. Vertical black dashed lines indicate the start and end of the one-minute load portion of the respiratory exercise. The high load, male time trace has the last 100 seconds removed due to one subject coughing which resulted in large muscle activation.

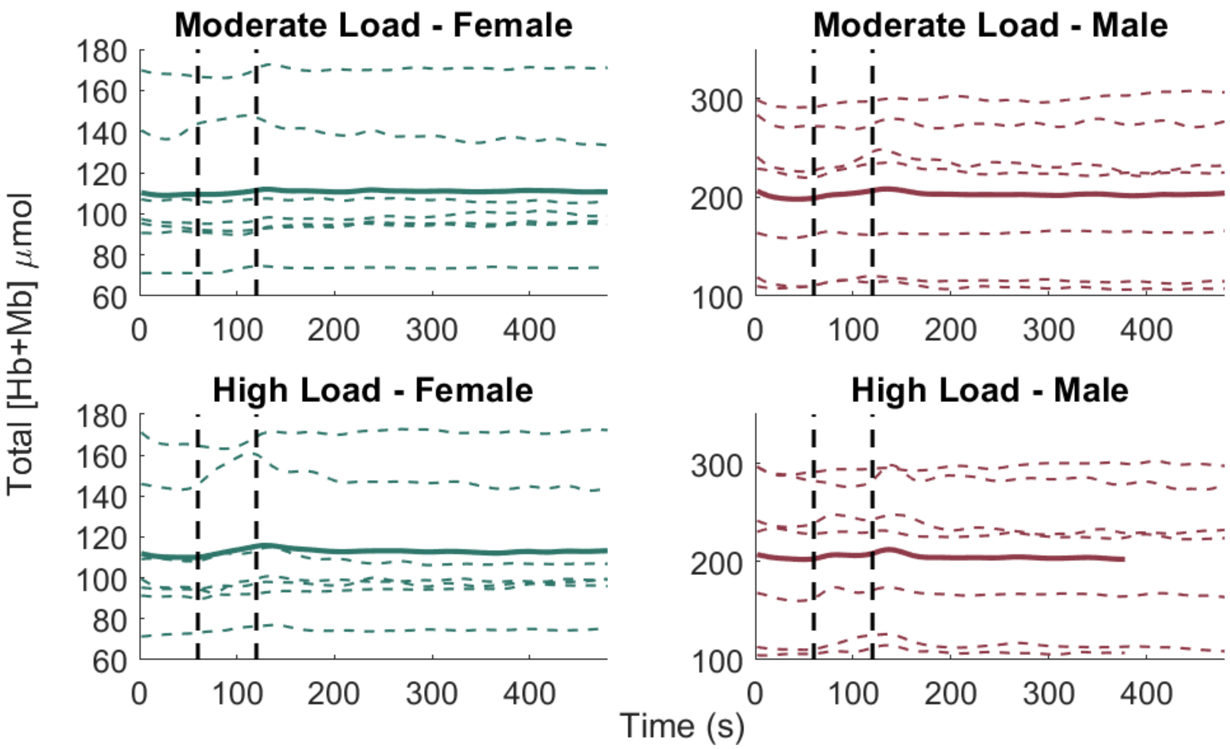

Figure S3. Filtered time traces of each subject and mean filtered time trace for each load and sex. Vertical black dashed lines indicate the start and end of the one-minute load portion of the respiratory exercise. The high load, male time trace has the last 100 seconds removed due to one subject coughing which resulted in large muscle activation.

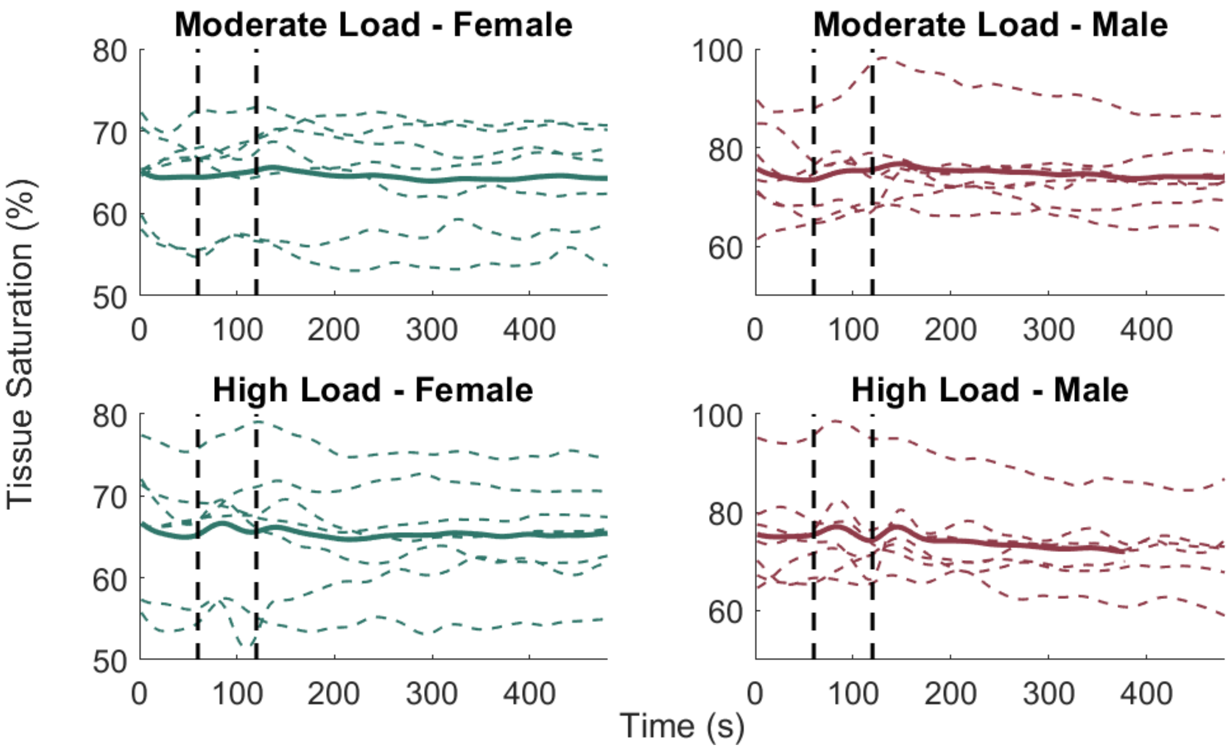

Figure S4. Filtered time traces of each subject and mean filtered time trace for each load and sex. Vertical black dashed lines indicate the start and end of the one-minute load portion of the respiratory exercise. The high load, male time trace has the last 100 seconds removed due to one subject coughing which resulted in large muscle activation.

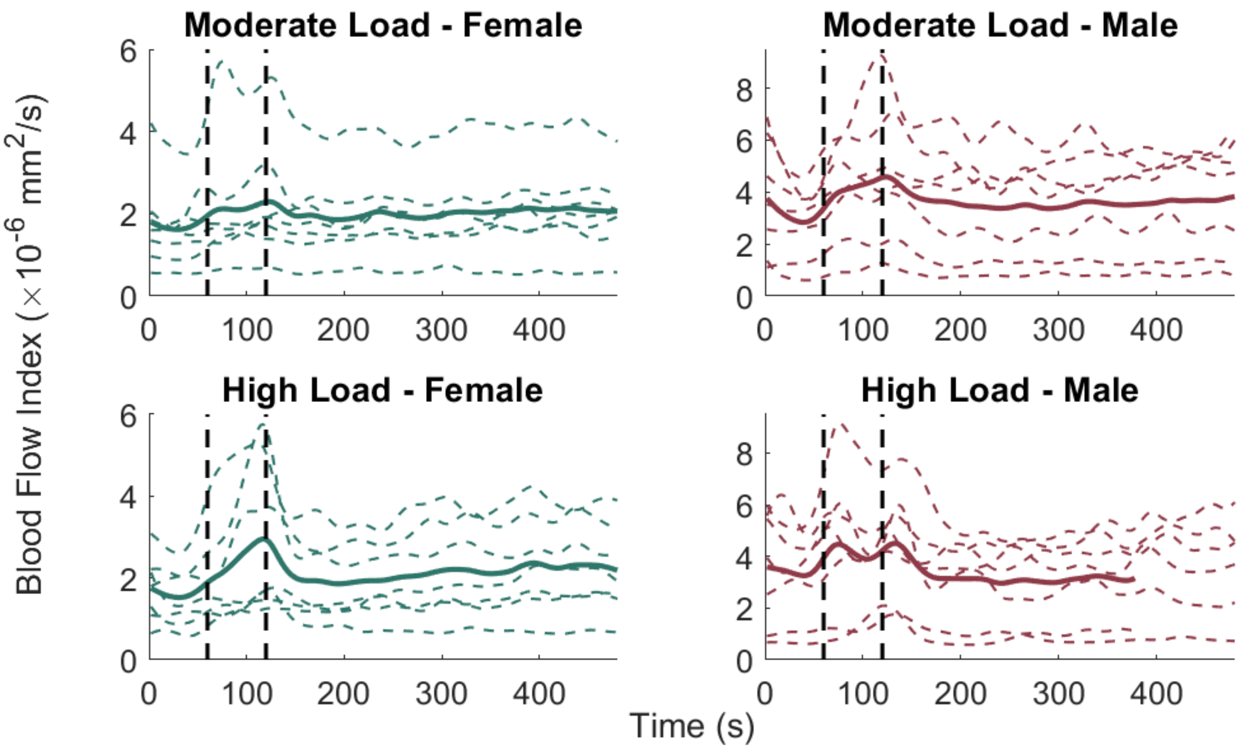

Figure S5. Filtered time traces of each subject and mean filtered time trace for each load and sex. Vertical black dashed lines indicate the start and end of the one-minute load portion of the respiratory exercise. The high load, male time trace has the last 100 seconds removed due to one subject coughing which resulted in large muscle activation.

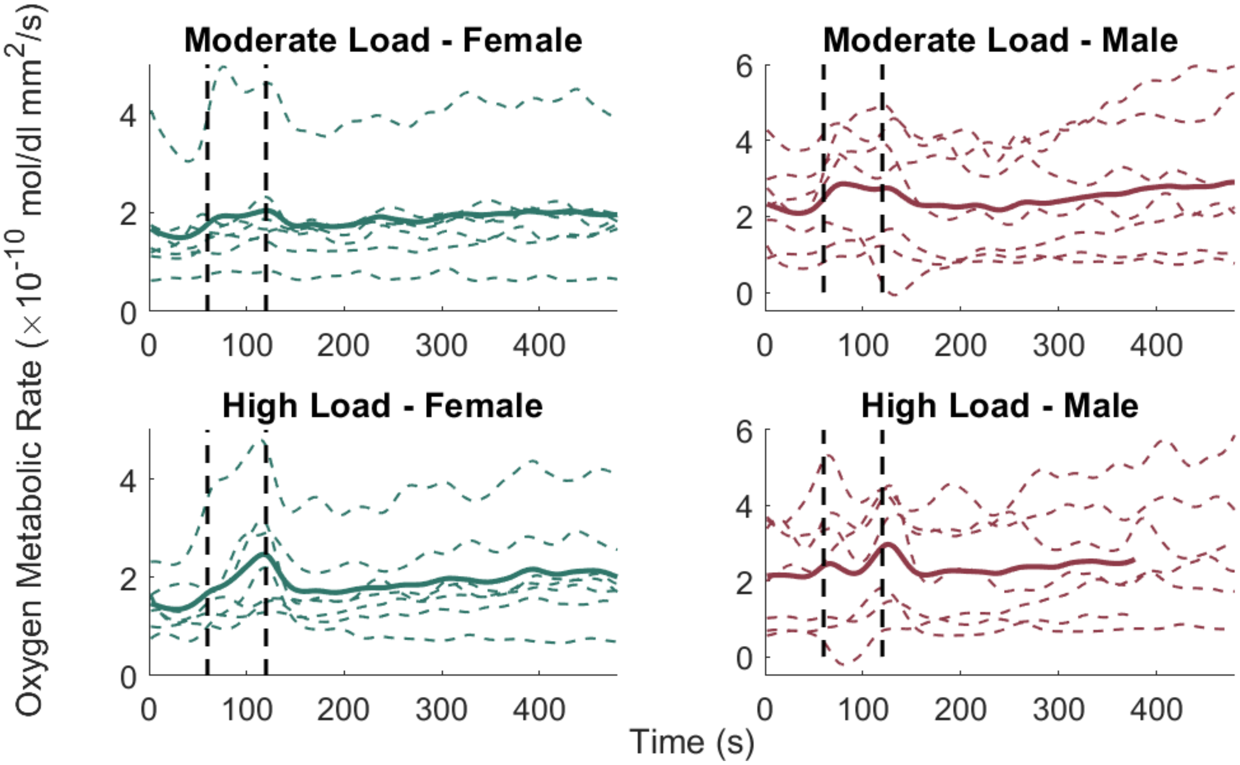

Figure S1. Filtered time traces of each subject and mean filtered time trace for each load and sex. Vertical black dashed lines indicate the start and end of the one-minute load portion of the respiratory exercise. The high load, male time trace has the last 100 seconds removed due to one subject coughing which resulted in large muscle activation.

Table S1. Mean ± standard distribution of the offset (sec) of the six measured muscle parameters for all sex, load, and region of activation combinations. Values were calculated only if more than two peaks/valleys were present in the region of activation.

		Female		Male	
		1st Region	2nd Region	1st Region	2nd Region
Moderate Load	Oxy [Hb+Mb]	17.3 ± 13.2	77.1 ± 40.6	23.3 ± 3.8	86.0 ± 31.8
	Deoxy [Hb+Mb]	–	58.3 ± 19.9	22.8 ± 8.1	118.0 ± 36.8
	Total [Hb+Mb]	27.3 ± 2.5	71.0 ± 8.0	23.6 ± 4.3	75.1 ± 4.5
	S_tO_2	16.7 ± 15.2	73.7 ± 23.9	24.0 ± 1.6	87.3 ± 33.6
	BF_i	16.0 ± 13.3	68.0 ± 18.1	19.7 ± 5.7	64.3 ± 5.8
	MRO_2	14.8 ± 12.7	67.0 ± 17.8	17.4 ± 7.1	66.7 ± 7.2
High Load	Oxy [Hb+Mb]	25.3 ± 3.4	70.6 ± 10.5	22.0 ± 4.2	80.3 ± 3.7
	Deoxy [Hb+Mb]	–	89.6 ± 29.4	21.3 ± 4.1	107.6 ± 53.7
	Total [Hb+Mb]	–	72.9 ± 15.2	–	74.6 ± 8.1
	S_tO_2	24.0 ± 3.3	84.4 ± 32.1	19.0 ± 4.6	105.7 ± 52
	BF_i	20.0 ± 12.8	60.6 ± 5.5	12.3 ± 7.4	69.4 ± 6.7
	MRO_2	22.7 ± 12.7	78.9 ± 47.2	12.7 ± 9.6	68.0 ± 6.0

Table S2. Mean ± standard distribution of baseline and absolute change of the six measured muscle parameters for all sex, load, and region of activation combinations. Values were calculated only if more than two peaks/valleys were present in the region of activation.

		Female			Male		
		Baseline	1st Region	2nd Region	Baseline	1st Region	2nd Region
Moderate Load	Oxy [Hb+Mb] (µmol)	71 ± 22	3.32 ± 2.96	3.76 ± 5.07	139 ± 50	6.13 ± 4.97	18.3 ± 15.7
	Deoxy [Hb+Mb] (µmol)	38 ± 10	–	-0.90 ± 2.17	50 ± 29	1.75 ± 6.8	-15.1 ± 13.0
	Total [Hb+Mb] (µmol)	109 ± 30	-1.11 ± 0.85	3.74 ± 2.82	189 ± 69	3.72 ± 2.36	7.64 ± 3.23
	S_tO_2 (%)	64 ± 5.2	1.53 ± 0.38	1.49 ± 3.12	75 ± 7.9	2.64 ± 2.06	4.22 ± 5.12
	BF_i ($x10^{-6}$ mm^2/s)	1.68 ± 0.93	0.72 ± 0.56	0.73 ± 0.56	2.82 ± 1.56	0.99 ± 0.44	1.81 ± 1.17
	MRO_2 ($x10^{-10}$ mol/dl mm^2/s)	1.55 ± 0.80	0.62 ± 0.53	0.54 ± 0.41	1.98 ± 1.02	0.73 ± 0.64	1.05 ± 0.66
High Load	Oxy [Hb+Mb] (µmol)	73 ± 25	1.76 ± 1.98	4.80 ± 7.04	144 ± 60	8.46 ± 10.2	13.7 ± 6.15
	Deoxy [Hb+Mb] (µmol)	37 ± 9.7	–	1.21 ± 3.56	47 ± 26	-7.33 ± 2.48	8.85 ± 11.5
	Total [Hb+Mb] (µmol)	110 ± 31	–	5.94 ± 5.41	192 ± 72	–	7.87 ± 4.48
	S_tO_2 (%)	65 ± 7.1	1.82 ± 1.22	0.32 ± 4.33	74 ± 9.3	3.03 ± 3.41	-1.33 ± 7.05
	BF_i ($x10^{-6}$ mm^2/s)	1.60 ± 0.70	0.88 ± 0.73	1.40 ± 1.20	3.06 ± 1.84	1.33 ± 1.25	1.23 ± 1.12
	MRO_2 ($x10^{-10}$ mol/dl mm^2/s)	1.40 ± 0.50	0.99 ± 0.49	1.13 ± 0.73	1.96 ± 1.26	1.00 ± 0.40	0.86 ± 0.65

Table S3. Mean ± standard distribution of respiration rate (Hz) for all load and sex combinations during baseline and both regions of activation.

		Baseline	1st Region	2nd Region
Moderate Load	Female	12.3 ± 1.2	11.7 ± 2.1	11.0 ± 0.8
	Male	13.1 ± 4.9	10.7 ± 2.1	10.4 ± 2.9
High Load	Female	10.9 ± 1.5	10.0 ± 2.1	10.7 ± 0.9
	Male	10.1 ± 3.3	9.3 ± 2.1	9.4 ± 2.0
